# Supplementary material for: Drosophila Males Differentially Express Small Proteins Regulating Stem Cell Division Frequency in Response to Mating
Source: J Dev Biol. 2025 Jun 23;13(3):21. doi: 10.3390/jdb13030021 (PMC12286032; doi:10.3390/jdb13030021)
Supplement: Supplementary file 1 [file jdb-13-00021-s001.zip › jdb-3590220 - Supplementary Data.pdf]

Table S1. DEGs in testes tips from non-mated and mated *OR* males.

Excel file, columns as indicated. Gene ID and abbreviations as in Öztürk-Çolak et al. (2024). DEGs (and one pseudogene) with significant q-values (<0.05, yellow) in both *wt* strains are marked in red, and blue, respectively.

Table S2. DEGs in testes tips from non-mated and mated *CS* males.

Excel file, columns as indicated. Gene ID and abbreviations as in Öztürk-Çolak et al. (2024). DEGs (and one pseudogene) with significant q-values (<0.05, yellow) in both *wt* strains are marked in red, and blue, respectively.

Table S3. MI^{GSC} counts from non-mated and mated males from different genetic backgrounds, as indicated.

Gene	BL Stock #	Crossed to	MI^{GSC} non-mated	MI^{GSC} mated
DEGs that increased MI^{GSC} in the control crosses but failed to increase MI^{GSC} when reduced from the germline				
anp	55385	*NG4*	23/391=5.9%	22/275=8%
			62/700=8.9%	54/775=7%
			31/578=5.4%	20/328=6.1%
		wt	16/319=5%	28/264=10.6%
			22/375=5.8%	37/266=13.9%
arc1/3	25954	*NG4*	29/469=6.2%	37/490=7.5%
			90/375=13.3%	46/389=11.8%
			18/340=5.5%	14/293=5%
			34/347=9.8%	37/337=11%
		wt	17/302=5.6%	27/346=7.8%
			34/303=11.2%	46/324=14.2%
			39/313=12.5%	90/278=32.4%
bbd	60483	*NG4*	30/425=7.1%	32/503=6.4%
			47/501=9.4%	28/398=7%
		wt	28/294=9.5%	37/294=12.6%
CG17242	55191	*NG4*	18/631=2.8%	16/655=2.4%
			20/493=4.1%	8/306=2.6%
		wt	43/423=10.2%	47/333=14.1%
dup99B	60069	*NG4*	26/379=6.9%	40/469=8.5%
			18/406=4.4%	15/450=3.3%
		wt	48/539=8.9%	66/461=14.3%
			8/240=3.3%	17/192=8.8%
sfp79B	60353	*NG4*	24/359=6.7%	21/401=5.2%
			21/309=6.8%	14/333=4.2%
			27/302=8.9%	28/285=9.8%
			28/349=8%	27/363=7.4%
		wt	32/347=9.2%	67/369=18%
			28/213=13.4%	34/166=20.5%
sfp93F	77337	*NG4*	31/723=4.3%	23/484=4.7%
			32/716=4.7%	25/490=5.1%
			50/514=9.7	31/339=9.1
		wt	21/373=5.6%	33/395=8.3%
			17/382=4.45%	38/381=10%
			35/303=11.5	51/290=17.6
RNAi against Signal Recognition Proteins				
srpr-beta	34011	*NG4*	tiny testes	tiny testes

		wt	17/256=6.6%	26/182=14.3%
srp9	34568	*NG4*	12/283=4.2%	13/259=5%
srp14	60428	*NG4*	tiny testes	tiny testes
		wt	22/254=8.7%	40/161=24.8%
srp54	30533	*NG4*	27/610=4.4%	23/557=4.1%
		wt	11/217=5.1%	21/169=12.4%
	55254	*NG4*	16/686=2.3%	24/696=3.4%
NG4		*wt*	30/327=9.1%	46/341=13.5%
			25/354=7.1%	48/366=13.1%
			13/434=3%	31/493=6.3%
		Accessory gland manipulations		
apc26Bx-Gal4		*UAS-DTI*	28/365=7.7%	32/285=11.2%
apc26Bx-Gal4		*wt*	43/365=13%	55/353=15.9%
UAS-DTI	35039	*wt*	43/389=11%	44/263=16.6%
prd^{Ketel}		prd^{Ketel}	13/243=5.4%	28/192=14.6%
			16/374=4.3%	39/374=10.4%
		wt	34/626=5.4%	42/515=8.2%

Gene names, Bloomington stock number for the RNAi lines, genotype crossed to, and resulting MI^{GSC} for non-mated and mated males are shown.

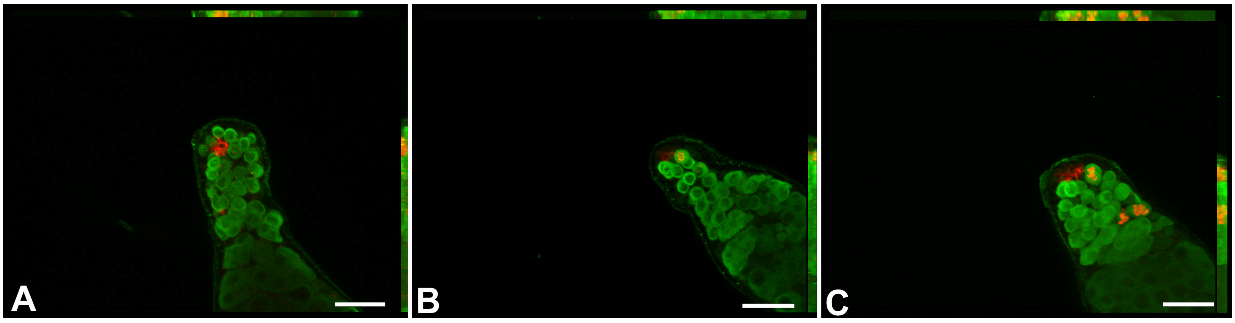

Figure S1. Original images used to rotate and crop the GSC images for Figure 2A-C. A-C) Immuno-fluorescence images of the apical testes regions from *OR* animals, stained with anti-Vasa to visualize the germline cells (green), anti-FasIII to label the hub cells (red) and anti-pHH3 to identify the cells in division (red). Scale bars 100µm.

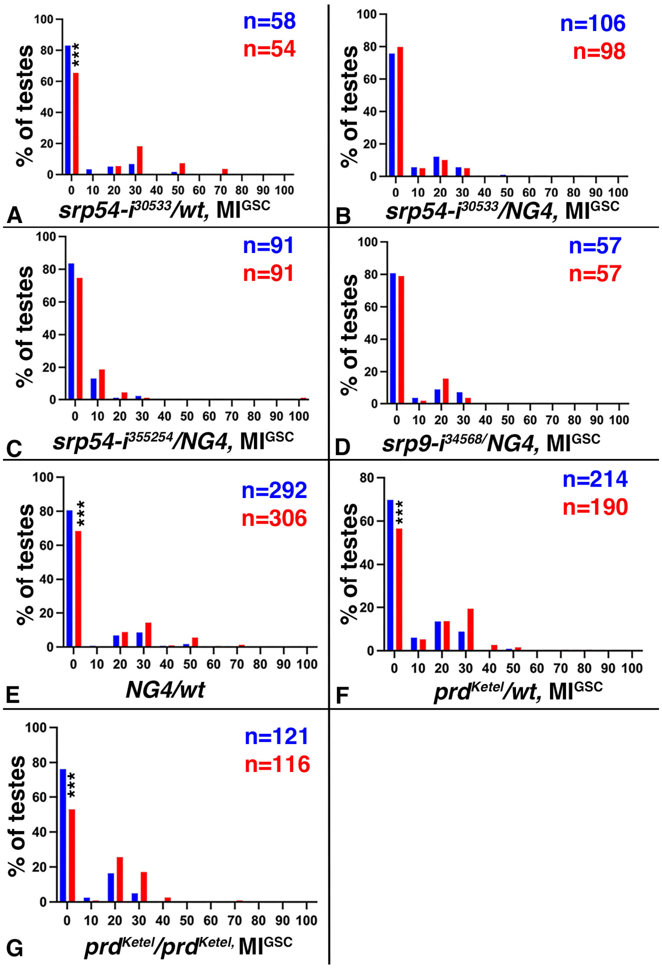

Figure S2. Frequency Distribution Graphs (FDGs) for animals with reduced *srps* and lacking accessory glands. X-axes: bins of ten based on individual MI^{GSC}, Y-axes: percentage of testes with that bin. Genotypes: (gene abbreviation-i(for RNAi)$^{\text{Bloomington stock number}}$) as indicated, color-coding as indicated, numbers of testes as indicated, asterisks: $p < 0.001$.

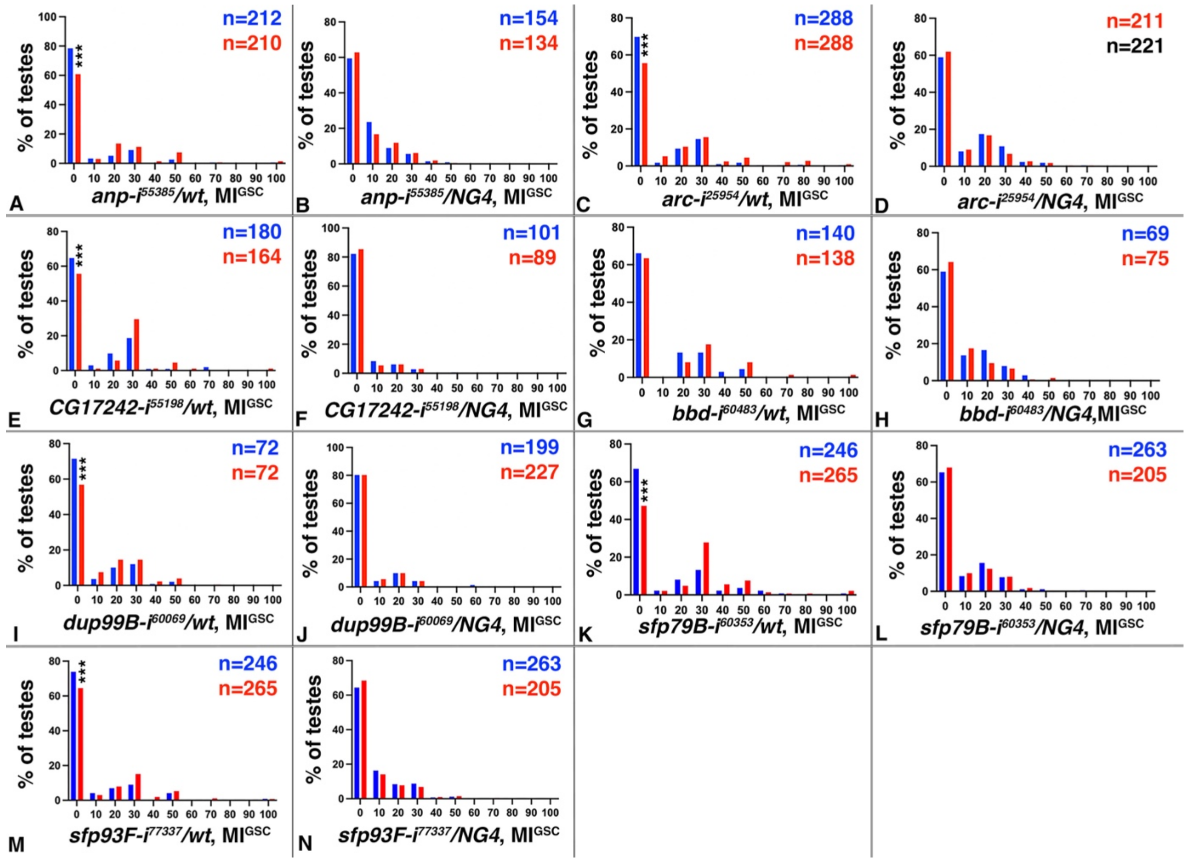

Figure S3. Frequency Distribution Graphs (FDGs) for animals with reduced small protein expression. X-axes: bins of ten based on individual MI^{GSC}, Y-axes: percentage of testes with that bin. Genotypes: (gene abbreviation-i(for RNAi)$^{\text{Bloomington stock number}}$) as indicated, color-coding as indicated, numbers of testes as indicated, asterisks: $p < 0.001$. (Note that *bbd-i/wt* did not show a significant reduction in testes with zero GSCs in division but had multiple testes in higher bins.)
